# Mpox and the Ethics of Outbreak Management: Lessons for Future Public Health Crises

**DOI:** 10.1111/dewb.70001

**Published:** 2025-07-18

**Authors:** Adetayo E. Obasa, Marietjie Botes, Anita Kleinsmidt, Ciara Staunton

**Affiliations:** ^1^ Registrar Research Support Office, Research and Internationalisation Development and Support, Faculty of Medicine and Health Sciences Stellenbosch University Cape Town South Africa; ^2^ Stellenbosch University Centre for Research on Evaluation Science and Technology Stellenbosch South Africa; ^3^ University of KwaZulu‐Natal School of Arts Durban South Africa; ^4^ University of the Western Cape, Faculty of Law Bellville South Africa; ^5^ Eurac Research Institute for Biomedicine Bolzano Italy

**Keywords:** epidemic, ethics, Mpox, pandemic, TRIPS

## Abstract

Mpox, first identified in captive monkeys in 1958 and recognized in humans by 1970 in the Democratic Republic of Congo, was historically confined to sporadic zoonotic outbreaks in Central and West Africa. These outbreaks, often driven by rodent‐to‐human transmission in resource‐limited settings, reflect persistent systemic health disparities. In recent years, mpox has also been reported in high‐income countries (including the United States, United Kingdom and Europe), underscoring its global health implications beyond traditionally endemic regions. This article examines the mpox outbreak through the lens of public health ethics, evaluating how core ethical frameworks—justice (encompassing equitable vaccine distribution and addressing health inequities), solidarity and respect for rights (including intellectual property considerations)—shape outbreak management strategies. These strategies are ensuring equitable access to vaccines and therapeutics amid intellectual property barriers, combating stigma and misinformation through transparent risk communication and fostering international solidarity in coordinating responses. The analysis highlights how neglect of these principles exacerbates existing disparities and undermines the effectiveness of interventions. Integrating ethical principles into outbreak responses is critical for building public trust, accountability and community resilience. By distilling lessons from the mpox response, this article contributes to ongoing debates in public health ethics and outbreak preparedness, arguing that ethically grounded approaches are essential for fair and effective management of future public health crises.

## 1 Introduction

Mpox is a zoonotic viral disease that was first identified in 1958 in a Danish laboratory, where it appeared among captive monkeys [1]. It was then diagnosed in a 9‐month‐old baby in 1970 in the Democratic Republic of Congo (DRC) [2]. For the next five decades, sporadic outbreaks occurred, and the virus became endemic in parts of Central and West Africa [3]. Despite its long presence in African communities, mpox remained a relatively neglected disease globally. This changed in 2022, when mpox cases began emerging in numerous countries outside its endemic regions in countries such as Canada, Portugal, Spain, the United Kingdom and the United States of America. The outbreak spread to over 100 countries, with more than 100,000 confirmed cases worldwide.

By December 2023, 2803 cases and 22 deaths had been reported across 10 African nations. On 14 August 2023, the World Health Organization (WHO) declared mpox a Public Health Emergency of International Concern (PHEIC) under the International Health Regulations. In a parallel move, the Africa Centres for Disease Control and Prevention (Africa CDC) declared mpox a Public Health Emergency of Continental Security (PHECS) the day before, marking a pivotal moment in Africa's efforts to tackle public health threats through unified action [4]. In 2024, a total of 16,291 laboratory‐confirmed cases, including 60 fatalities, have been reported across 19 countries, with most cases documented in the DRC, which accounted for 11,984 cases, followed by Burundi, with 2704 cases, and Uganda, with 1027 cases [5].

In managing outbreaks such as mpox, we must balance competing rights and interests, such as the public interest in stopping the spread of the virus, with the rights and freedoms of all individuals. Due to the globalized and interconnected world today, national responses are affected by those of other country and regions. This is important for access to diagnostic tools and treatment. Whether mpox has been ethically managed remains an open question thus far. Although efforts have been made to contain the outbreak, the global response has revealed weaknesses in addressing these ethical challenges [6].

During a disease outbreak, lack of transparency erodes public trust, compliance falters and the most vulnerable are left unprotected. An ethical approach is essential not only for the immediate containment of the disease but also for solidarity, shared accountability and resilience in the face of future public health threats [7]. The management of mpox‐type outbreaks requires a strong focus on ethics because public health responses have a direct impact on individuals rights, trust in the system and access to care [8]. Any response without strong ethical underpinning risks deepening inequalities and inequities. The response to the mpox outbreak has emphasized the necessity of thoroughly evaluating the ethical implications, particularly in contexts where marginalized populations face systemic inequities. It is crucial to consider how public health measures may inadvertently exacerbate these disparities. Addressing the global mpox outbreak requires careful attention to various ethical issues and considerations [9].

In this article, we provide an ethical reflection through the lens of public health ethics, to address epidemic and outbreak ethical dilemmas, emphasizing the importance of integrating ethical frameworks into outbreak management. We look beyond the status quo and underscore that it is crucial to embed ethical considerations at every stage of outbreak management to ensure a fair and effective public health emergency response.

## 2 Equitable Distribution of Vaccines and Treatment

Mpox vaccine distribution in Africa requires a framework grounded in justice, solidarity and transparency. These principles are essential for addressing historical inequities, building global cooperation and ensuring accountable and effective public health interventions [10]. Their application is not merely ethical but also practical, as they underpin the success of health programmes in contexts characterized by resource constraints, systemic barriers and diverse cultural dynamics.

### 2.1 Justice

Justice means fairness and the equitable distribution of benefits and burdens in society and healthcare [11]. Addressing systemic barriers and historical inequities, justice helps to mitigate health disparities and promotes equity, ensuring that life‐saving interventions are allocated based on need rather than wealth or geopolitical influence [12]. During the COVID‐19 pandemic, the African Vaccine Acquisition Task Team (AVATT) exemplified this principle by securing millions of vaccine doses for the continent and prioritizing vulnerable populations, demonstrating a commitment to equitable access and the protection of those most at risk and prioritized vulnerable populations [4]. In the context of mpox, justice would require us to prioritize regions like the DRC, where healthcare infrastructure is limited and access to essential resources is often constrained [13].

According to the World Health Organization (WHO), limited testing capacity continues to hamper mpox response efforts in the DRC. In 2024, only about 40% of suspected mpox cases were tested, an improvement from 9% in 2023, but still insufficient, given the scale of the outbreak [14]. Of the 5,160 confirmed cases, 25 deaths were recorded, resulting in a case fatality ratio (CFR) of 0.5%. In contrast, among the 21,835 suspected cases (tested and untested), the CFR was significantly higher at 3.3%, with 717 deaths reported. The low testing rate suggests that many infected individuals remain undiagnosed, allowing the virus to spread unchecked in remote and underserved regions.

This situation raises serious ethical concerns related to distributive justice, which requires that health resources be allocated fairly, especially to the most vulnerable [11]. In the DRC, systemic constraints such as weak laboratory infrastructure and a severe shortage of healthcare workers disproportionately affect rural and marginalized populations, who bear the highest burden of disease and yet have the least access to diagnosis and care. For example, the DRC spends only around $13 per capita on health, far below the sub‐Saharan African average [15]. The country has just 0.28 doctors and 1.19 nurses and midwives per 10,000 people, reflecting profound underinvestment in health system capacity [16]. By comparison, the United States allocates 17.7% of its GDP to healthcare, while the DRC spends only 3.5% [17]. Furthermore, the DRC ranks in the 18th percentile globally for effective universal health coverage, indicating that over 80% of countries outperform it in providing basic health services [18]. This stark inequity highlights not only a national failure to fulfil the demands of distributive justice but also the urgent need for global solidarity. While the international community must step up support to strengthen disease surveillance and laboratory infrastructure in high‐burden, resource‐limited settings, it is equally important that national governments prioritize health system reform. Without stronger local commitment, external assistance will not be sustainable. Addressing these gaps requires coordinated global and domestic efforts to promote ethical, equitable and resilient health systems [19].

### 2.2 Solidarity

Solidarity, within African ethical thought, can be understood as a compassionate and imaginative commitment to collective action that strengthens our inherent or cultivated connections, enabling us to thrive together [20]. Global health solidarity is a collective responsibility and the bedrock of global health security [21]. In the context of an outbreak, this will mean making available all necessary medical countermeasures such as vaccine production to all regions [22]. Recognizing that infectious diseases know no borders, a unified approach empowers all nations to build resilient health systems, enhancing global health security [23]. This strategy moves beyond traditional assistance models that risk perpetuating paternalism and dependency, focusing instead on mutual interdependence and respect for African autonomy. Solidarity ought to be rooted in a commitment to health equity, addressing systemic disparities and understanding health needs comprehensively, beyond just infectious diseases, to foster a balanced, sustainable and ethical global health landscape. Global health solidarity can bridge the gap between resource‐rich and resource‐poor settings, demonstrating a unified response to health challenges and reinforcing global equity in health interventions [20].

The initial global response to the mpox outbreak has mirrored the inequities observed during the COVID‐19 pandemic, particularly in vaccine distribution. Wealthier nations have secured substantial vaccine stockpiles, often exceeding their immediate needs, while low‐income countries, especially in Africa, face significant shortages [24]. Addressing these disparities requires a concerted effort to prioritize equitable vaccine distribution, including increasing manufacturing capacity in low‐income regions and ensuring timely access to medical resources. Without such measures, the cycle of inequality observed during the COVID‐19 pandemic is poised to repeat itself with mpox, hindering global health security.

### 2.3 Transparency

Transparency is a cornerstone of effective public health responses during outbreaks, as it fosters trust, enables timely interventions and ensures accountability. Transparency is fundamental to building trust and accountability in public health initiatives [21]. The WHO emphasizes that during public health emergencies, communicators must provide clear, accurate and timely information, even when all facts are not yet known. This approach helps maintain public trust and encourages compliance with health recommendations [25]. Public reporting, clear communication and open reporting on vaccine access and treatment safety, efficacy, availability and distribution criteria are essential to build stakeholders’ confidence [26, 27]. Africa CDC has played an important coordinating role in the mpox outbreak. It emphasizes transparency in its operations. The transparent public reporting of disease outbreaks and vaccination efforts is important in this context. They regularly update the public on the status of health emergencies and the effectiveness of interventions in a process followed by countries like the DRC, Nigeria and Burundi [28].

Effective epidemic control requires breaking transmission chains by ensuring that new cases do not arise from known contacts. Vaccines play a critical role in disrupting these chains; however, they are not a standalone solution. An effective response must include building local capacity in genomics research, vaccine development and prioritizing effective health communication and literacy initiatives in low‐ to middle‐income countries [29]. Health literacy and risk communication are essential components of this strategy, as they help build trust and foster community engagement. Sharing information in a way that resonates with diverse audiences—ranging from government officials and policymakers to lay members of the community—reduces fear and misinformation while promoting an appreciation for science [30]. For instance, Canada's unified messaging across political lines helped maintain public adherence to health guidelines [31]. New Zealand's former prime minister, Jacinda Ardern, was praised for her clear, empathetic and consistent communication during the COVID‐19 pandemic. Her regular updates, transparent decision‐making and use of simple language helped build public trust and compliance with health measures [32]. During the SARS outbreak in 2003, Taiwan's government implemented a robust communication strategy that included daily press briefings, transparent reporting of cases and clear guidelines for the public [33, 34]. This approach helped contain the outbreak and maintain public trust.

## 3 Intellectual Property and Vaccine Access: Challenges and Solutions

The World Trade Organization's Trade‐Related Aspects of Intellectual Property Rights (TRIPS) Agreement contains flexibilities intended to help countries address public health needs. Two key mechanisms are the TRIPS waiver and compulsory licensing. A TRIPS waiver is a collective agreement by WTO members to suspend certain IP obligations during a crisis. For example, in October 2020, India and South Africa proposed waiving enforcement of patents, trade secrets and other IP on COVID‐19 health products [35]. Over 100 nations supported this waiver as a crucial tool to overcome patent barriers and boost global vaccine supply [36]. The goal was to empower more manufacturers in developing countries to produce vaccines, medicines and diagnostics without fear of legal barriers. The United States also backed the concept in 2021, but the European Union and a few other wealthy members opposed it, blocking the consensus needed for adoption [37, 38, 39]. As a result, no comprehensive waiver was in place during the critical first 2 years of the pandemic. A watered‐down waiver was eventually agreed to in mid‐2022, limited only to vaccines and with eligibility restrictions, far too late to markedly improve vaccine equity. The waiver saga highlighted how political hurdles can undermine TRIPS flexibility's effectiveness in a timely pandemic response. The other major TRIPS flexibility is compulsory licensing (CL), which permits governments to authorize production or importation of a patented product without the patent holder's consent, typically with payment of royalties. In principle, TRIPS allows issuing a compulsory license, especially in emergencies, to facilitate access to essential medicines [40]. However, during COVID‐19, the CL mechanism proved cumbersome and inadequate for scaling up vaccine supply. Under TRIPS rules, compulsory licenses are mostly issued on a country‐by‐country and product‐by‐product basis, which would require many separate licenses to address a global need [40]. Although WTO rules (Art.31bis) permit exporting medicines made under a CL to countries lacking manufacturing capacity, this pathway has been used successfully only once (in 2007, to send HIV drugs from Canada to Rwanda) due to its complexity and political pressures on LMICs not to bypass patents [41]. Moreover, producing complex vaccines like mRNA or JYNNEOS (for mpox) requires technical know‐how that patents alone cannot provide and companies were reluctant to share proprietary knowledge. Thus, neither voluntary measures nor existing compulsory licensing provisions ensured timely, affordable COVID‐19 vaccine access in the global South.

## 4 From Covid‐19 to Mpox: Déjà vu in Vaccine Inequity

The inequities seen in COVID‐19 were tragically echoed in the 2022 mpox outbreak. Mpox was declared a Public Health Emergency of International Concern by the WHO in July 2022, but global vaccine distribution mirrored the earlier ‘haves and have‐nots’ pattern. The only WHO‐recommended mpox vaccine (Modified Vaccinia Ankara, sold as Jynneos or Imvamune) is produced by a single small manufacturer, with limited supply and a price of around $110 per dose [9]. Wealthy countries once again secured the lion's share: by August 2022, the United States had acquired over a million doses (roughly 80% of the global supply), while African nations had received zero doses via the market [42]. Africa's first small batch of 50,000 mpox vaccine doses arrived only as a donation from South Korea months into the outbreak. This disparity is especially stark, given that the virus had been endemic in parts of Africa for decades and African populations suffered the majority of mpox fatalities [42]. As Dr. Matshidiso Moeti of WHO/Africa noted in September 2022, ‘Africa is still not benefiting from either [mpox] vaccines or the antiviral treatments’, even as cases declined in the West due to vaccination [42]. The mpox response demonstrated that, despite lessons from COVID‐19, the international community still lacked effective mechanisms to ensure equitable vaccine access for outbreaks—a failure of both global solidarity and the current IP regime. Notably, the COVID‐19 TRIPS waiver (as adopted in 2022) did not cover mpox and no new waiver or widespread compulsory licensing effort emerged to address the crisis. The outcome was another instance of vaccine injustice.

## 5 Ethical Principles and Reflections

These twin experiences of mpox and COVID‐19 underscore the urgent need for reforms grounded in ethical principles of justice, solidarity and transparency. Justice suggests a fair distribution of life‐saving vaccines and treatments, so that one's chances of protection do not depend on nationality or wealth. The stark ‘vaccine apartheid’ seen in COVID‐19 and mpox violates the principle of justice and fuels preventable morbidity and mortality in poorer regions [43]. Solidarity entails that nations and corporations cooperate in the face of global health threats rather than prioritize profits or nationalist hoarding. Genuine solidarity would mean sharing technology and know‐how, donating or reallocating doses to where they are most needed and treating an outbreak anywhere as a threat to people everywhere [44]. International initiatives like COVAX tried to embody solidarity but were undermined by vaccine nationalism and insufficient support [45]. Transparency is equally critical: during COVID‐19, many bilateral vaccine deals and licensing agreements were secretive and decision‐making often excluded voices from the global South. Transparency in IP licensing, vaccine pricing and allocation would build trust and accountability, ensuring that promises of equitable access are verifiable and that any emergency measures (waivers or CLs) are implemented openly and efficiently. Building these principles into global health governance is essential to avoid repeating past failures. One promising avenue is the proposed WHO pandemic treaty (or ‘pandemic accord’) now under negotiation [46]. This treaty aims to strengthen pandemic prevention and preparedness, and it offers an opportunity to establish binding rules for equitable sharing of vaccines and technology in future outbreaks. Embedding waivers or expedited compulsory licensing provisions into such an accord—effectively pre‐authorizing the suspension of IP barriers during public health emergencies—would operationalize justice and solidarity. The treaty discussions have called for measures like transparent data sharing, local manufacturing support in LMICs, and reserve capacities for surge production of medical countermeasures [46]. Alongside legal changes, high‐income countries and pharmaceutical companies must be held accountable to ethical norms: profits and patent rights should not come before human lives in a global crisis. A reformed system, guided by the principle that no population should be left behind, would prioritize global health equity and dismantle the colonial‐era disparities that persist in health innovation and access.

## 6 Ethical Challenges of Stigmatization in the Mpox Outbreak

The outbreak was marred by two overlapping stigma narratives. The first cast mpox as an ‘African disease’, bolstered by news reports using imagery of African people that reinforced racist stereotypes. This framing violates ethical principles by perpetuating harm and inequity [47]. Singling out one region for blame diverted attention from the fact that viruses spread globally, violating principles of justice. The second narrative labelled mpox a ‘gay disease’ [48]. Since many early cases were among men who have sex with men (MSM), some commentary echoed AIDS‐era homophobia by blaming LGBTQ+ communities [49]. This stigma violated non‐maleficence by inflicting harm through scapegoating and discrimination, and it undermined beneficence by driving those at risk away from health services [50]. Anyone in close contact could catch mpox; focusing blame on one group was scientifically misleading and morally wrong. At its core, stigmatization contradicts the ethical obligation to treat all individuals with dignity while minimizing harm. Beneficence and non‐maleficence further require public health strategies to promote well‐being and avoid exacerbating harm through discriminatory language, imagery or policies [51].

Stigmatization had concrete consequences. Testing and treatment were hindered as people feared ostracization if diagnosed: some avoided clinics or even concealed their illness, and there were cases of patients being *denied* care due to bias [52]. A few individuals even fled quarantine because of stigma, potentially increasing the spread of the virus [53]. Such outcomes show how stigma undermined beneficence—people who needed care did not receive it, and public health efforts were impeded. Meanwhile, misinformation flourished in the climate of stigma. False beliefs that only Africans or gay men were at risk spread on social media, leading others to ignore their own risk and eroding public trust [50]. Communities that felt vilified were less likely to cooperate with health measures. Thus, stigma made the outbreak harder to control by weakening the trust and cooperation essential to public health.

Social media platforms amplified harmful, anti‐LGBTQ+ messaging that blamed the outbreak on gay individuals’ sexual behaviours [54]. Such stigma had profound consequences, including violent attacks, discriminatory practices and widespread mental health issues [55]. In countries like Nigeria, where homosexuality is criminalized, this stigma created barriers to testing, treatment and advocacy [56]. Media coverage and social media both spread and countered mpox stigma [50]. Early on, some coverage exacerbated fear with racist imagery or by overemphasizing gay sexual transmission, fuelling prejudice [49]. Social platforms likewise saw waves of homophobic and racist commentary. Social media remains the primary source of information among younger adults. While traditional outlets maintain credibility, their reach is dwarfed by social media figures. Health agencies and advocates stressed that anyone could catch mpox, promoting factual, stigma‐free information [50]. Experts implored communicators to avoid stigmatizing language that ‘casts blame on specific communities’, which only deters those who need care from seeking it (IDSA/HIVMA, n.d.). By sharing diverse patient stories and using inclusive language, responsible media helped counter fear and misinformation.

Scientists and health officials had a responsibility to counter misinformation and promote inclusive messaging. They took steps to correct harmful narratives. Experts urged the WHO to adopt the name ‘mpox’ to remove the racist connotations of “monkeypox” [9]. Major organizations also issued guidance against blaming any group: UNAIDS stressed that mpox is *not* a ‘gay disease’ and warned that stigma undermines outbreak control [50]. By providing accurate, stigma‐free information and engaging with affected communities, communicators upheld ethical principles and helped restore public trust.

## 7 Community Engagement as an Approach to Manage the Infodemic

According to the WHO, an infodemic—the rapid dissemination of both accurate and misleading information—poses a dual challenge of managing both the biological pathogen and misinformation [9]. The spread of misinformation violates principles of beneficence and non‐maleficence, leading to fear, confusion and mistrust that undermine public health efforts [57]. By actively partnering with communities, public health officials can repair trust that may have been eroded by the infodemic. Open dialogue—whether through community forums, social media questions and answers or advisory panels—showed respect for people's voices and concerns, making authorities more credible in the eyes of the public. In turn, greater trust improved compliance with interventions like vaccination and contact precautions.

Community engagement is critical for ethical management of an outbreak. Also ‘getting the health messaging right’ is particularly important for the success of this engagement [58]. For example, establishing community network links such as LGBTQ+ advocacy groups and local influencers can be leveraged in health campaigns, creating resilient channels for risk communication. Enhanced media literacy in the population will likewise pay dividends when confronting the next infodemic, be it in a pandemic or any public health emergency. Effective community engagement requires two‐way communication and collaboration with affected populations. Models such as Community Advisory Boards (CABs) enable dialogue between health authorities and communities, helping to address concerns and build trust [59]. Participatory health literacy campaigns promote understanding and informed decision‐making by tailoring messages to local languages and contexts [60]. Social listening tools track misinformation and identify trusted local figures—such as faith leaders or youth influencers—to deliver accurate information. In regions where stigma or misconceptions prevail, engagement should be tailored along class lines, audience values and belief, enhancing the credibility and uptake of information.

## 8 Conclusion

The mpox outbreak underscores the necessity for ethically grounded public health responses that balance individual rights with community well‐being. Future research should focus on developing context‐specific ethical frameworks addressing systemic barriers such as intellectual property constraints, infodemics, capacity building and stigma. In a world prone to pandemics, using inclusive language to enhance science communication and fostering deliberate community engagement are crucial. Advancing these areas will not only bolster outbreak preparedness and response but also contribute to a more just, inclusive and resilient global health landscape.

## Conflicts of Interest

The authors declare no conflicts of interest.
